# Online behavioural patterns for Coronavirus disease 2019 (COVID-19) in the United Kingdom

**DOI:** 10.1017/S0950268820001193

**Published:** 2020-06-05

**Authors:** M. D. Walker, M. Sulyok

**Affiliations:** 1 Department of the Natural and Built Environment, Sheffield Hallam University, Sheffield S1 1WB, UK; 2 Department of Pathology, Institute of Pathology and Neuropathology, Eberhard Karls University, Liebermeisterstr. 8, 72076 Tübingen, Germany; 3 Institute of Tropical Medicine, Eberhard Karls University, Wilhelmstr 27, 72074 Tübingen, Germany

**Keywords:** Coronavirus, COVID-19, infodemiology, respiratory disease, social media

## Abstract

The current coronavirus (COVID-19) pandemic offers a unique opportunity to conduct an infodemiological study examining patterns in online searching activity about a specific disease and how this relates to news media within a specific country. Google Trends quantifies volumes of online activity. The relative search volume was obtained for ‘Coronavirus’, ‘handwashing’, ‘face mask’ and symptom related keywords, for the United Kingdom, from the date of the first confirmed case until numbers peaked in April. The relationship between online search traffic and confirmed case numbers was examined. Search volumes varied over time; peaks appear related to events in the progression of the epidemic which were reported in the media. Search activity on ‘Coronavirus’ correlated well against confirmed case number as did ‘face mask’ and symptom-related keywords. User-generated online data sources such as Google Trends may aid disease surveillance, being more responsive to changes in disease occurrence than traditional disease reporting. The relationship between media coverage and online searching activity is rarely examined, but may be driving online behavioural patterns.

The Internet is now the favoured source of healthcare information by the general public [1]. The moniker ‘Dr Google’ has even entered our lexicon to denote the use of this search engine by those seeking online medical advice; often done prior to contacting trained medical professionals in person. Infodemiology is where such digital and online-generated data is used for epidemiological research purposes and to inform public health decisions [2]. Previous studies have examined the relationship between online search traffic and disease occurrence [3]. Influenza has been a favoured choice for such research [4]. Websites such as Google Trends offer a useful source of real-time data, possibly better reflecting disease occurrence than traditional and slower disease reporting through official channels [5].

However, such studies rarely examine factors, often country specific, affecting such online activity. Typically studies simply describe patterns between disease occurrence and search volumes, without attempting to explain them, or utilising such data for further modelling [3]. The Coronavirus disease 2019 (COVID-19) pandemic provides a unique opportunity to investigate patterns in online user activity on a novel disease, and examine how these relate to media coverage within a specific country. The progression of COVID-19 in the U.K. is well documented; with initial cases being reliably recorded by official bodies and reported by the news media. The magnitude of public interest in COVID-19 is unprecedented, meaning trends in online search traffic were likely to be robust and reliable. Few studies have attempted to relate online behaviour patterns to media reporting about a health condition.

Google Trends [6] indexes volumes of searching upon a specific search topic against a benchmark index of 100 to provide a relative search volume (RSV). This maximum figure is allocated to the date upon which online activity was greatest in the specified period. The RSV was downloaded from Google Trends for a variety of keywords related to the disease, including ‘Coronavirus’ (virus), ‘handwashing’ (search term) and ‘face mask’ (search term). Data were used from the date of the first confirmed case on 31 January 2020 until when reported cases numbers peaked on 12 April 2020.

Keyword choice depended on country-specific factors relevant to the U.K. The name officially recognised by WHO is Coronavirus Disease 2019 [7]; the RSV for ‘Coronavirus’ (virus) was used for analyses as this is the name used in U.K. government press releases and by the National Health Service (NHS) (Supplementary Table S2). Data on Coronavirus ‘Virus’, was used as this includes data allowing for spelling variations and assesses semantic meaning of searching. ‘Handwashing’ (search term) was purposely chosen as being the term used in a Department of Health media campaign run in March 2020 [8]. Google does not provide data for ‘handwashing’ as a topic. Similarly, ‘face mask’ is the wording used in official press releases and thus was chosen for analyses. Data on ‘face mask’ as a ‘search term’ was used as it correlated better with case numbers than that for ‘topic’ (Supplementary Table S3). The symptom keywords, ‘cough’ (search term) and ‘temperature’ (topic) are those listed in NHS guidance for the general public [9]. ‘Fever’ (search term) was a Google-generated suggestion when searching for symptoms. Where possible data for ‘topic’ was used in preference to that for ‘search term’. Data on related similar keywords to those chosen were compared and also correlated with confirmed case numbers (Supplementary Table S3).

Data on the number of confirmed cases for the entire U.K. were obtained from the European Center for Disease Control for the corresponding time periods [10]. Data were analysed with R version 4.0.0. Firstly, contemporaneous Spearman's rank correlations were performed on data for the periods from the date of the first confirmed case on 31 January 2020 up to 12 April 2020 when the number of confirmed cases peaked in the U.K.; this provides a logical end point to the initial phase of the outbreak. Previous studies have found stronger correlations where case data were time lagged [11–13]. Thus, next using the R cross-correlation function, data were time lagged from −35 to +35 days and the resulting correlation examined. Where peaks in RSV occurred, events related to the disease outbreak were sought in the news media and government press releases.

Figure 1(a) shows the RSV for ‘Coronavirus’ against the daily confirmed number of cases for the U.K. The pattern of the RSV mirrored the number of confirmed cases; but visually appears to precede them. The peak RSV occurred on 16 March. The contemporaneous correlation between ‘Coronavirus’ RSV and confirmed case number was strong (*ρ* = 0.77, *P* ≤ 0.001). Peaks in RSV were visually apparent (A, 31 January. B, 10 February. C, 28 February. D, 5 March 2020. E, 12 March. F, 16 March and G, 22/23 March). These could be related to specific events in the progression of the disease in the U.K. reported in government press releases and the news media (Supplementary Table S2).

**Fig. 1.**
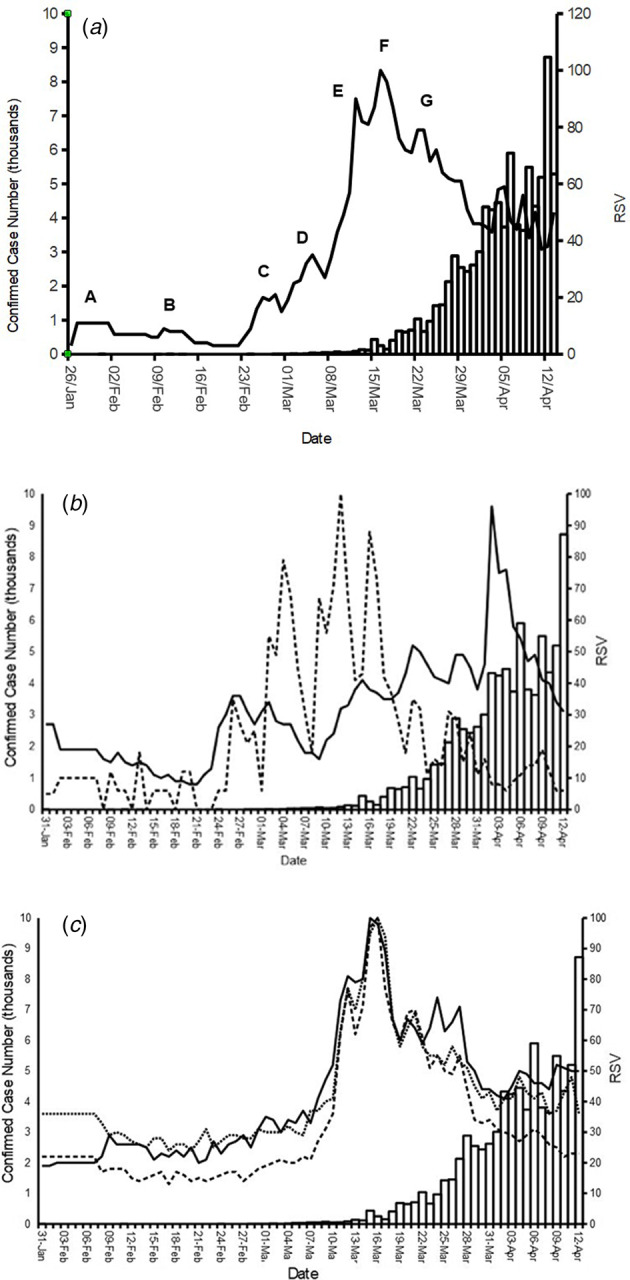
RSV compared with confirmed case numbers of Coronavirus (COVID-19) for keywords: (a) ‘Coronavirus’: solid line. Confirmed case numbers: Columns. Key to events: A: CMO confirms imported coronavirus case. B: Chief Medical Officer for England announces further cases. C: U.K. based locally acquired cases. D: First U.K. death, handwashing media campaign. E: Guidance to self-isolate for vulnerable. F: Guidance to avoid social contact, closing of retail and hospitality outlets. G: PM instruction to stay at home; ‘Lockdown’. (b) ‘handwashing’: dashed line, and ‘face mask’: solid line, (c) ‘fever’: solid line, ‘temperature’: dotted line, and ‘cough’: dashed line.

Figure 1(b) shows levels of the RSV for ‘face mask’ and ‘handwashing’ against confirmed case numbers. The RSV for ‘Face mask’ peaked on 4 April 2020. The RSV was strongly contemporaneously correlated with confirmed case numbers (*ρ* = 0.83, *P* ≤ 0.001). ‘Handwashing’ RSV peaked on 12 March, with lesser peaks on 5 March and 16 March. The RSV on ‘handwashing’ was poorly correlated with cases (*ρ* = 0.33, *P* = 0.0037). The RSV on the symptom keywords are similar to that observed for ‘Coronavirus’ RSV. The maximum RSV for both ‘cough’ and ‘temperature’ occurred on 16 March, and for ‘fever’ on 15 March. The similarity in RSV between the three symptom keywords is visually apparent (Fig. 1(c)). Each symptom proved strongly contemporaneously correlated to case numbers; ‘cough’ (*ρ* = 70, *P* ≤ 0.001), ‘temperature’ (*ρ* = 0.70, *P* ≤ 0.001) and ‘fever’ (*ρ* = 0.76, *P* ≤ 0.001).

The results of cross correlation using time lagged data suggest that the trends observed in RSV occurred prior to those observed in confirmed cases (Supplementary material S1). The cross-correlation function which permits time lagging of data provides the Pearson product coefficient. The RSV for ‘Coronavirus’ was most strongly correlated with a time lag of −20 days (*r* = 0.76), ‘handwashing’ at −25 days (*r* = 0.70), and ‘face mask’ at −4 days (*r* = 0.75). Similarly for the three symptoms examined; ‘temperature’ −20 days (*r* = 0.71), ‘cough’ −21 days (*r* = 0.75) and ‘fever’ −19 days (*r* = 0.74).

Comparison of the RSV of the keywords examined with that of close alternatives showed generally close similarity in RSV pattern, and produced similar levels of correlation (Supplementary Material S3, S4, S5). However, exceptions did occur. The RSV for ‘SARS-CoV2’ did not follow that for ‘Coronavirus’. Also, the RSV of ‘face mask’ (topic) did not follow that of other face mask keywords. The RSV of these, and ‘hand washing’ (search term), were notably less well correlated with case numbers than the other keywords (Supplementary Table S3).

Online behaviour data, such as that obtainable from Google Trends, have been used in recent studies of infectious diseases similar to COVID-19. Notably, the relationship between social media usage and the number MERS-CoV cases in Korea was examined [11]. Another study examined the relationship between Google Trends data and reporting on MERS-CoV in the WHO disease outbreak news [14]. Similar studies examining online behaviour patterns in relation to the current COVID-19 outbreak are being conducted. Recently, a study using linear regression and long short-term memory (LSTM) models attempted to predict COVID-19 incidence using Google Trends data for Iran [15]. Another examined Internet patterns in Italy, finding increasing interest in topics such as face masks and the symptoms of COVID-19 [16].

Other studies have suggested that patterns in RSV precede disease occurrence and reporting. A study currently in press also found that increases in RSV precede corresponding patterns in case numbers [13]. However, that study, using data from multiple countries, performed analyses before peaks in cases had been reached. It also only looked at a single search term and made no comparison between them. Similarly, a study examining Google searching and a MERS-CoV outbreak in Korea found that levels of online user activity preceded corresponding levels of laboratory confirmed cases [11]. The authors speculated that patients may have used Internet resources prior to seeking medical help. Another study examining RSV and Coronavirus in Taiwan found stronger correlations when case numbers were time lagged [12]. However, that study only used −3 to +3 day time lagging; additionally case numbers were small.

The preceding peaks in RSV before case numbers is most striking for the symptom keywords examined. This could well be the result of those experiencing such symptoms initially seeking online advice, as suggested by Shin et al. [11]. The peaks in RSV may thus suggest initial symptom development; the later corresponding peak in case number may be due to progression in the severity of illness, warranting medical attention some days later, with corresponding official recording. However, possibly more likely are that RSV peaks are related to the media and official output that occurred mid-March; the authors note that the peak RSV for ‘Coronavirus’ and the three symptoms examined occurred on the date upon which retail and hospitality outlets were recommended to close.

A possible relationship between keyword RSV and news and media output is most likely with ‘handwashing’. A major handwashing media campaign began on 4 March 2020 [8] (Supplementary Table S2). This campaign received widespread media coverage and may account for the patterns in RSV observed. A recent cross-country study suggested a relationship between the RSV of a handwashing-related search term and COVID-19 case numbers [17]. However, as the results presented here illustrate, caution is needed as slightly different keywords produce different patterns and strengths of correlation, even within a single country (Supplementary Table S3 and Fig. S4). This highlights the need to consider multiple keywords, and consider which are most relevant to the country being studied.

This study illustrates the importance of using justifiable country-specific search terms when examining Internet-based data sources. Such country-specific factors, such as the handwashing media campaign in the U.K., may be influencing online user behaviour, but are rarely considered. The time frame over which data are examined can influence patterns seen and also need justification. A limitation of this study is that routine testing of all suspected Coronavirus cases slowed from 13 March in the U.K., meaning case numbers may be higher than officially reported after this date. Another limitation is that RSV for symptom keywords are not COVID-19 specific; those with other illnesses may also use these terms when searching for online information.

The connection between RSV and confirmed case numbers at the beginning of a disease outbreak could be of importance in the surveillance of conditions where the situation is developing rapidly and up-to-date information about disease progression is required. User-generated Internet-based data potentially offer more time-sensitive information than is provided by standard surveillance. The interplay between online behaviour, media reporting and actual reported disease occurrence appears complex; these are topics requiring further study. Such knowledge could be of importance when planning public healthcare information campaigns, aiding in the optimal allocation of energies and resources. Should media coverage prove to drive online behaviour on specific healthcare matters then this would have implications regarding the timing of such information campaigns. Media efforts could be timed at the moment when most effective at initiating public action. Further studies examining online behaviour patterns should consider using multiple search terms and relating patterns in RSV to specific events concerning progression of disease in a single country.

## Supplementary material

For supplementary material accompanying this paper visit http://dx.doi.org/10.1017/S0950268820001193. click here to view supplementary material
