# Supplementary figures and images for: Online behavioural patterns for Coronavirus disease 2019 (COVID-19) in the United Kingdom
Source: Epidemiol Infect. 2020 Jun 5;148:e110. doi: 10.1017/S0950268820001193 (PMC7306408; doi:10.1017/S0950268820001193)

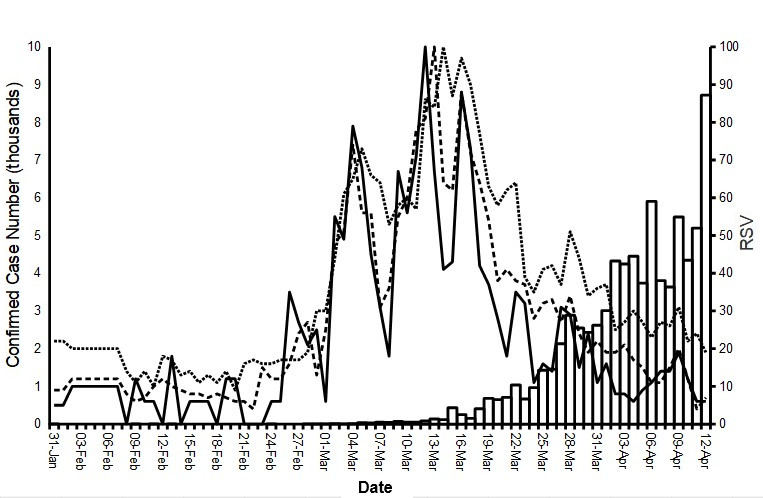

Supplement: Supplementary file 1 [file S0950268820001193sup001.zip › S0950268820001193sup001.jpg]

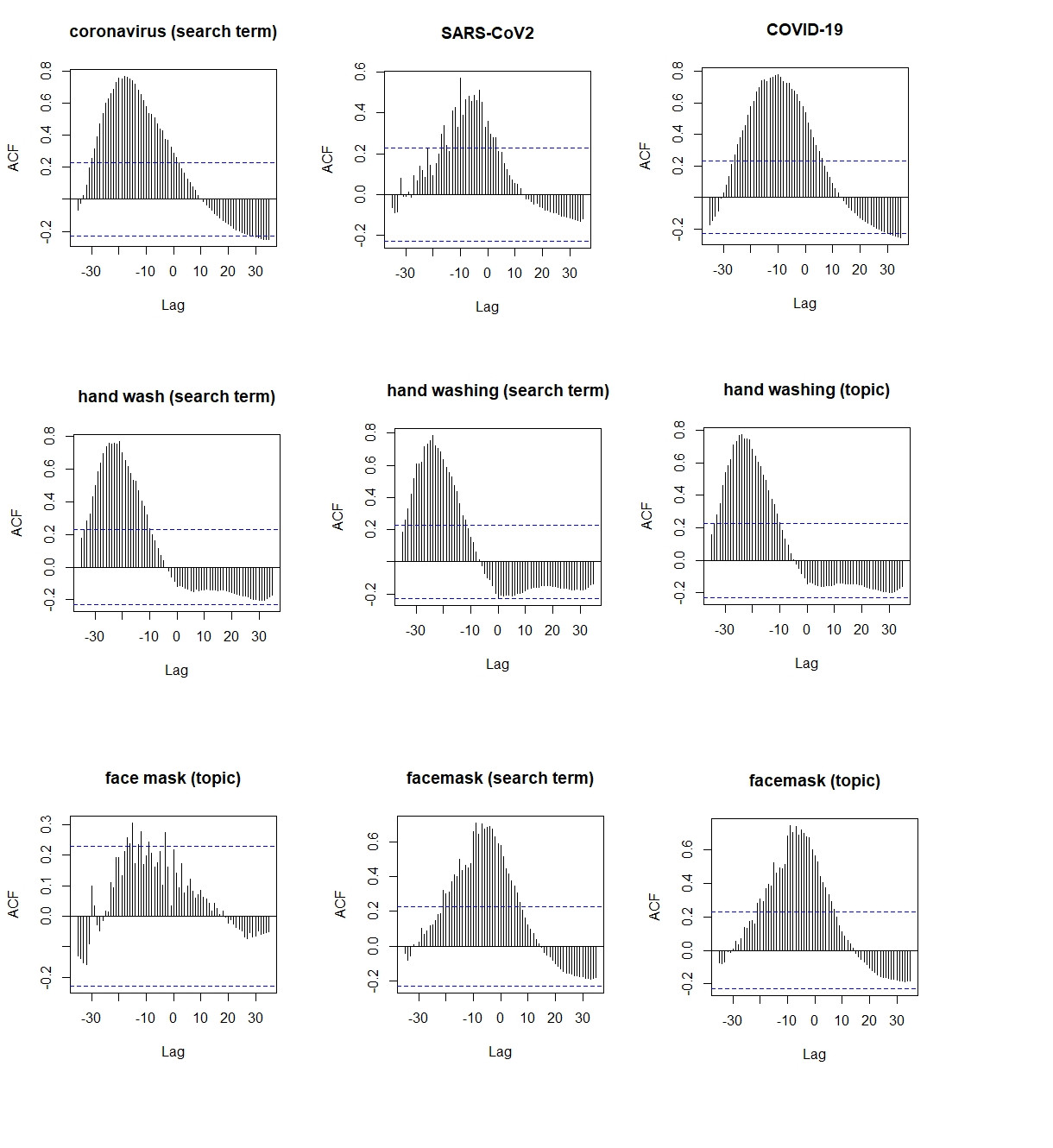

Supplement: Supplementary file 1 [file S0950268820001193sup001.zip › S0950268820001193sup002.jpg]

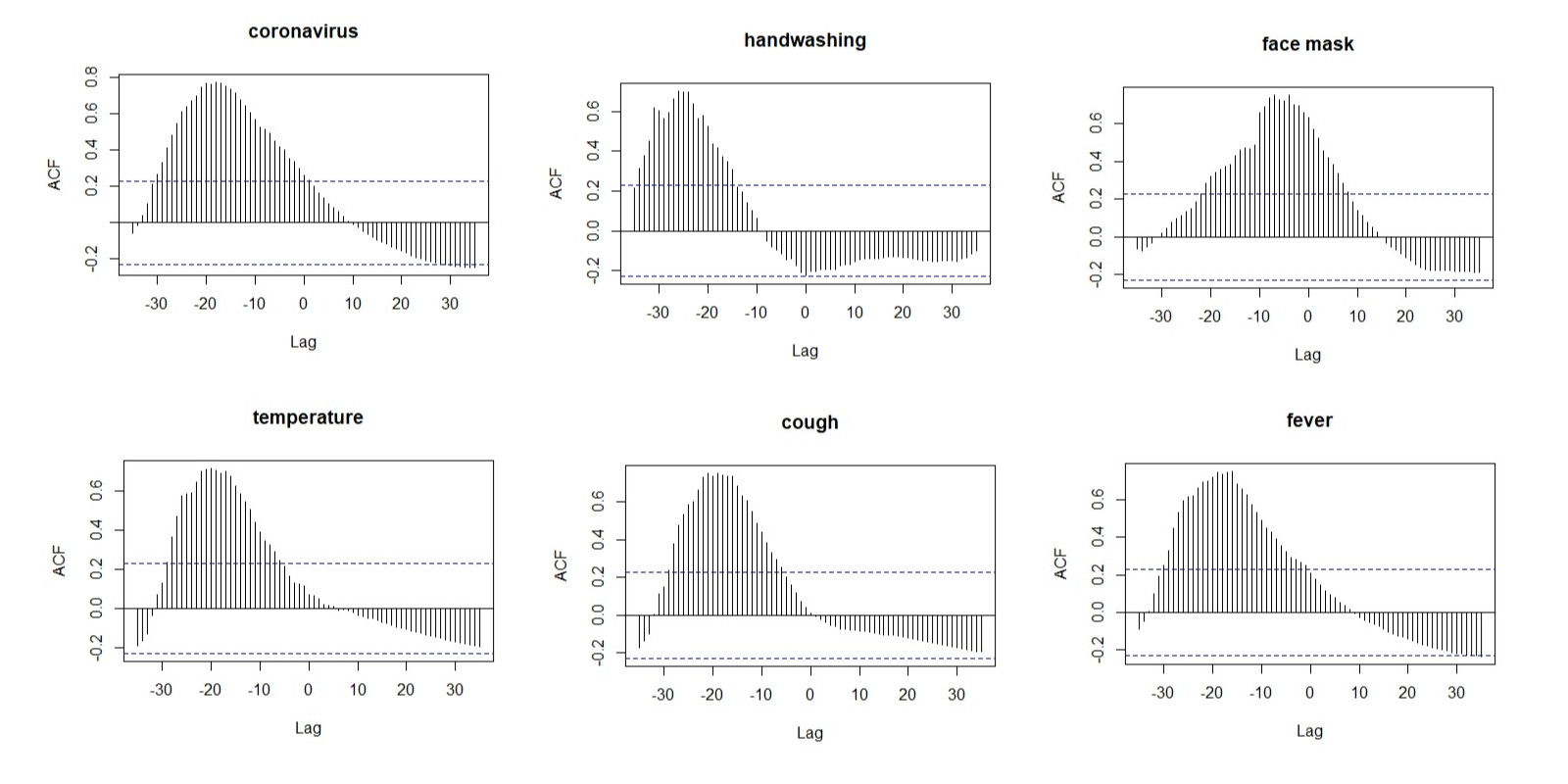

Supplement: Supplementary file 1 [file S0950268820001193sup001.zip › S0950268820001193sup005.jpg]

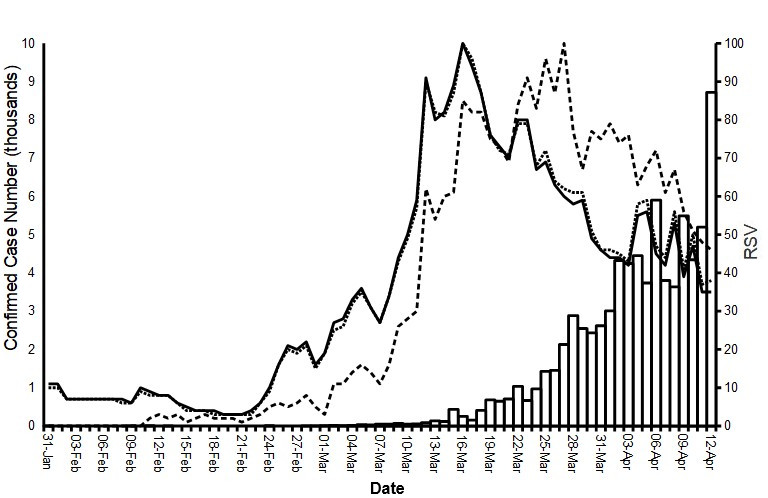

Supplement: Supplementary file 1 [file S0950268820001193sup001.zip › S0950268820001193sup006.jpg]

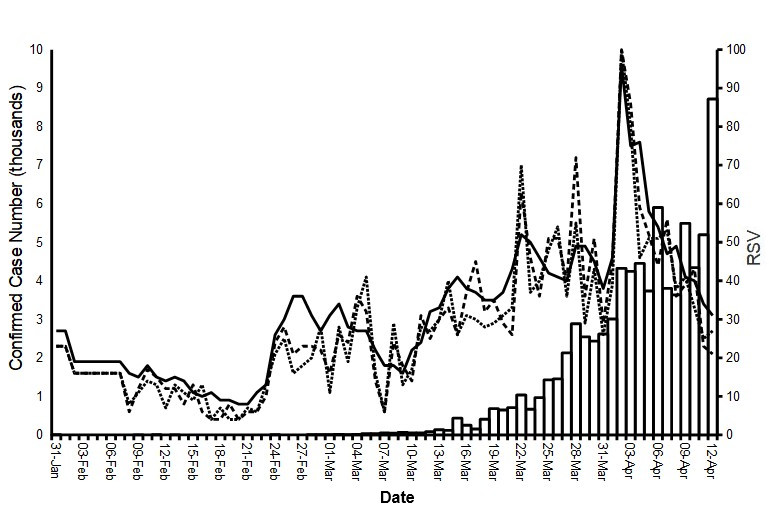

Supplement: Supplementary file 1 [file S0950268820001193sup001.zip › S0950268820001193sup007.jpg]
