# Supplementary material for: Online behavioural patterns for Coronavirus disease 2019 (COVID-19) in the United Kingdom
Source: Epidemiol Infect. 2020 Jun 5;148:e110. doi: 10.1017/S0950268820001193 (PMC7306408; doi:10.1017/S0950268820001193)
Supplement: Supplementary file 1 [file S0950268820001193sup001.zip › S0950268820001193sup003.doc]

**Supplementary Material**

**'Epidemiology and Infection'**

**Manuscript Title**: **Online behavioural patterns for Coronavirus disease 2019 (COVID-19) in the United Kingdom**

**Names of Authors: MD Walker^1^ & M Sulyok^2,3^**

**Supplementary Table S2:** Selected events related to the peaks in GTD. Press releases from gov.uk, BBC and selected media organisations.

| **GTD 'Peak'** | **Date** | **Event** | **Source** | **Link** |
| --- | --- | --- | --- | --- |
| A | 30 to 31 Jan | WHO declares global emergency.  Imported cases: CMO confirms cases of coronavirus in England  Repatriation of U.K. citizens from China.  Dramatic increase in face mask sales | BBC  gov.uk  BBC  The Independant  BBC | https://www.bbc.co.uk/news/world-51318246  <https://www.gov.uk/government/news/cmo-confirms-cases-of-coronavirus-in-england>  https://www.bbc.co.uk/news/uk-51318691  https://www.independent.co.uk/news/world/asia/coronavirus-news-live-uk-china-outbreak-symptoms-wuhan-evacuation-latest-a9312476.html#post-1184019879  https://www.bbc.co.uk/news/uk-wales-51299570 |
| B | 11 to 12 Feb | Chief Medical Officer for England announces 4 further coronavirus cases | gov.uk  BBC  BBC | https://www.gov.uk/government/news/cmo-confirms-fourth-case-of-coronavirus-in-england  https://www.bbc.co.uk/news/uk-51447761  https://www.bbc.co.uk/news/uk-england-sussex-51468813 |
| C | 26/28/29 Feb | Health Secretary's statement to Parliament: 26 February  Locally acquired cases reported: CMO for England announces a new case of novel coronavirus: 28 February 2020  Coronavirus: Latest patient was first to be infected in UK. .  Coronavirus passed on within UK. | Gov.uk  Gov.uk  BBC  Northern Echo | https://www.gov.uk/government/speeches/covid-19-health-secretarys-statement-to-parliament  https://www.gov.uk/government/news/cmo-for-england-announces-a-new-case-of-novel-coronavirus-28-february-2020  https://www.bbc.co.uk/news/uk-51683428  https://www.thenorthernecho.co.uk/news/national/18270758.coronavirus-passed-within-uk-first-time-20th-case-confirmed/ |
| D | 4 to 5 Mar | First U.K. death of patient with COVID-19  Coronavirus: Woman in 70s becomes first virus fatality in UK  Public Health England Handwashing campaign, published 4^th^ March  NHS Handwashing Promotion Video (Accessed from 'Metro' newspapers) | Gov.uk  BBC  Gov.uk  NHS | https://www.gov.uk/government/news/cmo-for-england-announces-first-death-of-patient-with-covid-19  https://www.bbc.co.uk/news/uk-51759602  https://www.gov.uk/government/news/public-information-campaign-focuses-on-handwashing  https://metro.co.uk/video/nhs-release-official-hand-washing-advice-against-coronavirus-2123306/ |
| E | 12 to 13 Mar 20 | PM statement informing those with symptoms to self isolate  Government guidance to self isolate if expericing symptoms |  | https://www.gov.uk/government/speeches/pm-statement-on-coronavirus-12-march-2020  https://www.gov.uk/government/publications/covid-19-stay-at-home-guidance |
| F  PEAK Coronavirus RSV | 16 to 17 Mar 20 | Prime Minister statement to avoid social contact  Statement by Health Secretary for vulnerable to self isolate and all to avoid social contact.  Renewed Government guidance to self isolate if expericing symptoms  Closing of pubs, bars and cafes |  | https://www.gov.uk/government/speeches/pm-statement-on-coronavirus-16-march-2020  https://www.gov.uk/government/speeches/controlling-the-spread-of-covid-19-health-secretarys-statement-to-parliament  https://www.gov.uk/government/news/new-guidance-for-households-with-possible-covid-19-infection  https://www.bbc.co.uk/news/uk-51917562 |
| G | 22 to 23 Mar | Prime Minister Statement enforcing stay at home only leave for essential reasons. 'Lockdown' begins/  BBC report on regulations |  | https://www.gov.uk/government/speeches/pm-address-to-the-nation-on-coronavirus-23-march-2020  https://www.bbc.co.uk/news/av/uk-52012581/coronavirus-you-must-stay-at-home-boris-johnson-orders |
