# Supplementary material for: Online behavioural patterns for Coronavirus disease 2019 (COVID-19) in the United Kingdom
Source: Epidemiol Infect. 2020 Jun 5;148:e110. doi: 10.1017/S0950268820001193 (PMC7306408; doi:10.1017/S0950268820001193)
Supplement: Supplementary file 1 [file S0950268820001193sup001.zip › S0950268820001193sup004.doc]

**Supplementary Material**

**'Epidemiology and Infection'**

**Manuscript Title**: **Online behavioural patterns for Coronavirus disease 2019 (COVID-19) in the United Kingdom**

**Names of Authors: MD Walker^1^ & M Sulyok^2,3^**

**Supplementary Table S3:** Correlation between alternative keywords and confirmed case number.

|  | **Correlation of RSV with**  **confirmed case numbers ρ (*p*)** |
| --- | --- |
| **'Coronavirus' related keywords** | |
| Coronavirus 'search term' | 0.77 (< 0.001) |
| SARS Co-V2 | 0.47 (< 0.001) |
| COVID-19 | 0.84 (< 0.001) |
| **'Face mask' related keywords** | |
| Face mask (topic) | 0.22 (0.064) |
| Facemask (search term) | 0.81 (< 0.001) |
| Facemask (topic) | 0.8 (< 0.001) |
| **'Handwashing' related keywords** | |
| Hand wash (search term) | 0.56 (< 0.001) |
| Hand washing (search term) | 0.39 (0.0005) |
| Hand washing (topic) | 0.56 (< 0.001) |
